# A cross-sectional study of the feasibility of pharmacy-delivered harm reduction services among people who inject drugs in Xichang, China

**DOI:** 10.1186/s12889-015-2236-x

**Published:** 2015-09-14

**Authors:** Yi Yang, Carl A. Latkin, Rongsheng Luan, Cui Yang

**Affiliations:** Department of Social Medicine and Health Administration, School of Administration, Chengdu University of Traditional Chinese Medicine, 1166 Liutai Avenue, Wenjiang District, Chengdu, 611137 China; Department of Health, Behavior and Society, Johns Hopkins Bloomberg School of Public Health, 2213 McElderry St. 2nd FL, Baltimore, MD 21205 USA; Department of Epidemiology and Biostatistics West China School of Public Health, Sichuan University, Chengdu, Sichuan Province 610041 China

## Abstract

**Background:**

HIV prevalence is high in Liangshan, China (1.1 %). In 2012, people who inject drugs (PWID) in Xichang, the capital city, contributed to 60.0 % of the HIV infections. The goal of the current study was to examine the feasibility of implementing pharmacy-delivered harm reduction services (PDHRS) for PWID.

**Methods:**

Face-to-face structured interviews with 403 PWID included questions on PWID’s experiences of syringe services and their specific experiences, acceptance, and potential usage of PDHRS.

**Results:**

There were some reports of harassment/bad treatment from pharmacists (12.2 %) and police (17.6 %). Non-prescription syringe sales (NPSS) from pharmacies in single piece were the main source (82.1 %) of syringes. 72.5 % of PWID reported visiting 31.5 % of the identified pharmacies. Most (74.7 %) PWID disposed of their used syringes by throwing them away. Only one PWID brought used syringes back to a pharmacy in the past 30 days. Half of the PDHRS, such as printed materials about HIV, Hepatitis C and STIs; risk reduction services; (16.9 %) and sharps container to dispose of syringes (0.2 %) were offered by a few pharmacies (<20 % for each service). The acceptance rates among PWID toward currently offered services were high (≥91.1 %). All potential PDHRS were acceptable by most (68–95.3 %) PWID, and correspondingly 67–94.5 % of PWID reported they would use each service if offered.

**Conclusions:**

NPSS from pharmacies provided many PWID in Liangshan with new syringes. However, disposal of used syringes was problematic. At the time of investigation, half of 16 assessed PDHRS were already available in pharmacies in Xichang. PWID were ready to use all the potential PDHRS and14 of 16 PDHRS were feasible to provide. HIV testing kits may be available in pharmacies in the future. Many pharmacy-delivered harm reduction services are feasible and acceptable among PWID in Xichang, China.

## Background

The first indigenous human immunodeficiency virus (HIV) cases in China were reported among heroin users in the Yunnan province in 1989 [1]. The overall HIV prevalence (0.058 %) in China remains low, but HIV prevalence among people who inject drugs (PWID) remains high [2]. Due to country-wide responses [3–6], by the end of 2011, Methadone Maintenance Treatment (MMT) (738 clinics covering 13.3 % of the total PWID) and Needle Exchange Program (NEP) (over 900 sites delivering more than 12 million clean syringes annually) have been scaled up in China [4, 7, 8]. Syringe distribution rates (per PWID per year) in China are greater than the global average, including levels in the United States and Russia [8]. HIV prevalence among PWID has been decreasing from 12.0 % in 2005 [3], 9.3 % in 2009 to 6.4 % in 2011 [4]. In certain areas, such as Liangshan Yi Autonomous Prefecture (Liangshan), Sichuan province, sharing syringes and other injection equipment are still common among PWID [5, 6, 9]. Between March 2004 and December 2012, HIV prevalence among PWID from 11 methadone clinics in Liangshan was found to be 25.4 % [7].

In 2012, China accounted for approximately 20 % of the global numbers of PWID [10, 11]. Sichuan province ranked 5th in the number of PWID in China [2]; most are from Liangshan [12, 13]. Liangshan has been part of the major drug trafficking routes in China for more than 100 years, and heroin use is common in Liangshan [14]. HIV incidence in Liangshan increased dramatically, accounting for 81.5 % of the total HIV/AIDS cases in the past 5 years [15]. According to the Asian Epidemic Model, PWID accounted for 52.3 % of the HIV infections in Liangshan in 2013, and are anticipated to be responsible for 39.1 % by 2020 [16].

Providing sterile syringes to PWID is a cost effective method of HIV prevention [17]. NEP and pharmacies are the major sources of sterile syringes [18]. Yet perceived discrimination [19], internalized stigma [20], and lack of stigma management strategies [21] impede PWID from using formal health services such as NEP [20, 22, 23]. Internationally, community pharmacies have been providing services such as non-prescription syringe sales (NPSS), HIV-testing, vaccinations, educational materials, and coupon syringe programs. Other services are potentially available in pharmacies, such as providing methadone, naloxone for overdose treatment, safer injection training programs, containers to dispose of used syringes, on-site disposal, testing for hepatitis, and directly observed therapy for PWID [18, 21, 24–38]. However, PWID have reported problems accessing available harm reduction services. Refusals to sell syringes and demands for picture identification or prescriptions have been documented even when purchasing syringes is legal and there is no official requirements for providing identification or a prescription [18, 29, 30, 35–37].

In China, syringes are considered medical devices. Pharmacies that sell syringes are required to obtain a “Medical Devices Operation Enterprises permit”, which is issued by the provincial bureau of the Food and Drug Administration (FDA) and supervised by the Prefecture FDA [39]. In Guangxi Province, distribution of pharmacy vouchers for syringes, ampoules of sterile water for injection, and condoms has been previously tested [40]. A survey among 324 PWID from detoxification centers in Yunnan Province documented that their major sources of syringes were pharmacies and health clinics [41]. Between 2008 and 2011, NEP among 19 counties in Yunnan had been implemented at pharmacies, clinics, and through peer educators [42].

NPSS to PWID in Liangshan have not been reported in previous literature, and it was unclear whether PWID would utilize potential PDHRS. Xichang, the capital of Liangshan, has a population is more than 600,000 [6]. In 2012, the official estimated number of PWID was 2250, and drug use contributed to 60 % of HIV infections [23]. The HIV prevalence among PWID in Xichang was found to be 11.3 % [43] in 2002, 17.8 % in 2004 [44], and 18.0 % in 2012 [23]. There is one MMT clinic, three MMT outpatient service sites, and three NEP sites. In 2012, there were more than 400 pharmacies. The goal of the present study was to examine the feasibility of a range of PDHRS for PWID in Xichang.

## Methods

This study was a cross-sectional study design, and the protocols were approved by both institutional review boards (IRBs) from Johns Hopkins Bloomberg School of Public Health (JHSPH) and the West China School of Public Health (WCSPH). The survey was developed as part of a multisite study on the feasibility of using pharmacies for PWID’s health services [23]. The protocol and questionnaire were initially written in English. The questionnaire was translated into Chinese, reviewed both by the US and China study team, and piloted. The survey took approximately 30 min to administer. The local collaborator, Xichang Skin Disease and Prevention Center (XSDPC), is a government agency responsible for HIV/AIDS prevention and control. The research team selected Xichang methadone clinic and detoxification center to recruit eligible PWID. The study inclusion criteria for PWID were: 1) 18 years of age or over and 2) had injected drugs within the past 30 days. From April to May, 2012, investigators from WCSPH and staff from XSDPC were trained as interviewers and conducted face-to-face structured interviews. Snowball sampling methods were used. After completing their interviews, PWID were encouraged to inform other PWID about the study. A total of 403 PWID were recruited from Xichang detoxification center (66.7 %), MMT clinic (15.6 %), communities (7.4 %), MMT outpatient service sites (6.7 %), and rehabilitation center (3.5 %). As participants volunteered for the study and came to the clinics for the interviews, response rates could not be calculated.

Before completing an interview, eligible participants provided informed consent, and 50 yuan (~USD 8) compensation was provided after completing the surveys. Condoms and safe injection supplies were provided as well as appropriate referrals. The survey questions included demographic characteristics, self-reported HIV status, methods of obtaining and disposal of syringes, experiences of syringe services in pharmacies, and the perceptions and potential utilizations of 16 potential PDHRS. The 16 PDHRS included: printed materials about HIV, Hepatitis C, and sexually transmitted infection(STI) risk reduction; blood pressure check; vaccinations for Hepatitis A and B; information about how to prevent abscesses; overdose medication (naloxone); sharps container to dispose of syringes; a written medical, drug treatment, and social service referrals; free condoms; HIV testing; training or information about how to inject safely; training or information about preventing overdoses; syringe exchange; disposal of used syringes; directly dispensing methadone; and free syringes. The response options toward potential PDHRS were binary (yes or no).

SPSS 22.0 (IBM, USA) was used for analyses. For univariate analysis, range and median for interval variables and frequencies for nominal variables were conducted. Fisher’s exact test was used for comparing the frequencies of receiving and giving used syringes among PWID and *P* < 0.05 as a level of statistical significance.

## Results

The characteristics of 403 participants are presented in Table 1. Almost all (95.8 %) PWID had been in prison/jail or forced drug treatment; 71.0 % reported that they had been tested for HIV, and among them 70.6 % said they were HIV negative, 14.7 % HIV positive, and 14.7 % did not know their status. The earliest test among the participants was reported in 1994. Overall, 41.7 % were HIV negative, 8.7 % HIV positive, 8.7 % had been tested but did not know their status, and 29.0 % had not been tested.

**Table 1:** Characteristics of PWID enrolled in Xichang, China 2012 (*n* = 403)

Characteristic	median(range)
Age, (years)	35(17–55)
Living in Xichang, (years)	28(1–51)
Education, (years)	8 (0–16)
Income in last month, (Chinese Yuan)	2000(0–30,000)
	No. (%)
Male Sex	353(87.6)
Ethnic group	
Han	213(52.9)
Yi	180(44.7)
others	10(2.5)
Currently enrolled in any kinds of school, college, a vocational or training program	3(0.7)
Stable housing	367(91.1)
Martial statues	
Married	187(46.4)
Single	116(28.8)
Divorced	86(21.3)
Widowed	7(1.7)
Separated	6(1.5)
Other	1(0.2)
Live arrangements	
With parents	173(42.9)
With sex partners	117(29.0)
Alone	87(21.6)
Others	25(6.2)
Employed	234(58.1)
Own a cell phone	336(83.4)

Abbreviations: People who inject drugs (PWID)

In the past 30 days, 2.7, 19.4, and 77.9 % of PWID, respectively reported “always”, “at least once”, and “never” using a used syringe; 3.5 % “always”, 25.3 % “at least once”, and 71.2 % “never” passed their syringe to someone else to use after they had used it. It is interesting to note that PWID reported receiving fewer used syringes than they distributed (Fisher’s exact test, *p* < 0.01).

### Receiving and disposing used syringes

Most (72.0 %) PWID agreed with the statement that “it was easy to get new sterile syringes”, and a large percentage (81.1 %) reported having purchased a syringe in a pharmacy in the last 30 days, with a median of six syringes for regular purchase. The most frequent (74.7 %) method for syringe disposal was throwing syringes away, and 24.3 % reported that they left syringes where they injected. Only 1 PWID brought used syringes to a pharmacy (Table 2).

**Table 2:** Obtaining and disposing of syringes in the Last 30 days among PWID from Xichang, China 2012 (*n* = 403)

Sources of syringes			Syringes disposal	
Source	No. (%)	Syringes usually obtained median(range)*	Source	No. (%)
Pharmacy	327(81.1)	6(1–180)	Throwing syringes away	302(74.7)
Medical facility	93(23.1)	6(1–500)	Left syringes where they injected	98(24.3)
Someone else obtain syringes from pharmacy	48(11.9)	3(1–100)	Buried or burned syringes	72(17.9)
NEP	36(8.9)	10(2–100)	Brought syringes to a NEP	20(5.0)
Syringe seller/drug dealer	25(6.2)	4.5(1–50)	Put syringes in a sharps container, or soda or laundry bottle, then threw it away	20(5.0)
Bought from a friend, relative, or acquaintance (PWID or non-PWID)	10(2.5)	5(2–20)	Gave syringes to someone else to take to a NEP for them	18(4.5)
Received free from a friend, relative or acquaintance (PWID or non-PWID)	10(2.5)	5(1–25)	Gave syringes to an outreach worker or peer educator	10(2.5)
From someone who said they got them at a NEP	6(1.5)	10(1–50)	Brought syringes to a pharmacy	1(0.2)
From an outreach worker	2(0.5)	21.5(8–35)	-	
Used prescription for syringes because of diabetic or other medical condition	1(0.2)	20(20–20)	-	

Abbreviations: People who inject drugs (PWID) needle exchange program (NEP)

*: median and range were calculated only among PWID who obtained syringes from the source

### Experiences of buying syringes from pharmacies

Most (82.1 %) PWID reported having purchased a syringe in a pharmacy in the last 12 months, over half (63.5 %) reported that the minimum number of syringes that they could buy in a pharmacy in Xichang was one, and 59.3 % of participants reported the price for one syringe was one Chinese Yuan (~$0.16). Almost two-thirds (64.5 %) of PWID agreed with the statement “it’s generally hard for me to go to the pharmacy during the hours they are open.” A similar proportion (65.2 %) preferred to buy syringes at a pharmacy where they buy over-the-counter (OTC) or prescription drugs; 43.8 % reported that they used pharmacies in the same neighborhood/region where they live. There were 309 (76.7 %) PWID who named the pharmacies where they bought syringes most of time, and 124 unique pharmacies were identified. One pharmacy was frequently visited by 23(5.7 %) PWID and 31.5 % of the 124 identified pharmacies were visited by more than one PWID (Table 3).

**Table 3:** Frequently visited pharmacies for syringes purchase among PWID from Xichang, China 2012 (*n* = 309)

No. of PWID naming a specific pharmacy	No. of pharmacies named	Percentage of pharmacies named (%)	No. of PWID	Percentage of PWID^€^
1	85	68.5	85	21.1
2	15	12.1	30	7.4
3	4	3.2	12	3.0
4	2	1.6	8	2.0
5	2	1.6	10	2.5
6	4	3.2	24	6.0
7	2	1.6	14	3.5
8	3	2.4	24	6.0
10+	7	5.6	102	25.3
total	124	100.0	309	76.7

Abbreviations: People who inject drugs (PWID) €: *n* = 403

When buying syringes in pharmacies in the last 12 months, 18.6 % of PWID reported that their requests were declined, and 12.2 % were harassed or treated poorly at the pharmacy (Table 4). Some (17.6 %) PWID reported that they had been stopped or poorly treated by the police near a pharmacy.

**Table 4:** Syringes purchase experiences in pharmacies among PWID from Xichang, China 2012 (*n* = 403)

	Occurred in the last 12 months (No. (%))	Frequency in the past 30 days (median(range))^£^
Declined to sell syringes	75(18.6)	3(0–100)
Harassed or treated badly at the pharmacy	49(12.2)	3(0–30)
Asked what the syringes will be used for	30(7.4)	3(1–30)
Refused to sell ‘single’ syringes	12(3.0)	2(0–10)
Asked to sign a log-book or required written personal information	3(0.7)	2(1–3)
Asked to show a photo identification card	3(0.7)	1.5(1–2)

*PWID* refers to people who inject drugs

£: median and range were calculated only among PWID who experienced in the last 12 months

### Pharmacy-delivered services

Among the 16 listed potential services in the pharmacies, eight were currently offered by ≤16.9 % of pharmacies. The most commonly offered service was printed materials about HIV, Hepatitis C, and STI risk reduction services (16.9 %), and the least common were sharps containers (0.2 %) and written medical or social service referrals (0.2 %). The acceptance rates were high among services currently offered (≥91.1 %). The eight services that were associated with harm reduction, such as HIV testing and free syringes, were not currently offered in pharmacies. The 16 potential harm reduction services were acceptable to most PWID (68–95.3 %), and most (67–94.5 %) PWID reported that they would use the service if offered (Table 5).

**Table 5:** Availability and feasibility for PDHRS among PWID in the last 12 months from Xichang, China 2012 (*n* = 403)

Service category	Services currently available (No. (%))		Potential services (No. (%))	
	Offered/availabe	Accepted/used^€^	Good idea to offer	Personally use if offered
Printed materials about HIV/Hepatitis C/STIs risk reduction	68(16.9)	65(95.6)	384(95.3)	381(94.5)
Blood pressure check	45(11.2)	41(91.1)	369(91.6)	367(91.1)
Training/information about preventing overdoses	10(2.5)	10(100.0)	350(86.8)	347(86.1)
Training/information about how to inject safely	5(1.2)	5(100.0)	337(83.6)	334(82.9)
Training/information about how to prevent abscesses	3(0.7)	3(100.0)	353(87.6)	353(87.6)
Overdose medication (naloxone/narcan)	2(0.5)	2(100.0)	278(69.0)	277(68.7)
Sharps container to dispose of syringes	1(0.2)	1(100.0)	281(69.7)	276(68.5)
A written medical or social service referral	1(0.2)	1(100.0)	301(74.7)	302(74.9)
Free condoms	0	0	346(85.9)	340(84.4)
Vaccinations for hepatitis A/B	0	0	335(83.1)	326(80.9)
HIV testing	0	0	317(78.7)	308(76.4)
Free syringes	0	0	325(80.6)	322(79.9)
To dispose of used syringes	0	0	290(72.0)	285(70.7)
To exchange used syringes for new ones	0	0	287(71.2)	285(70.7)
A written drug treatment referral	0	0	280(69.5)	280(69.5)
Directly dispensing methadone	0	0	274(68.0)	270(67.0)

Abbreviations: *PDHRS* Pharmacy-Delivered Services, *HIV* human immunodeficiency virus, STIs, sexually transmitted infections

€: *n* = offered or available in the last 12 months

## Discussion

At the time of this study, government funded detoxification center, MMT, and NEP services were available, but no government funded HIV prevention funds provided to pharmacies for harm reduction services in Xichang. The first available harm reduction service reported in this sample of PWID was HIV testing, which occurred in 1994, 5 years after the first large national HIV outbreak among PWID in Yunnan [1]. As almost all (95.8 %) PWID had been in prison or jail or forced drug treatment where HIV testing is mandatory, it was expected that over 95 % should have been tested. However the self-report HIV testing rate was only 71.0 %. Therefore, it is probable that HIV positive status was under-reported [23]. In this study, syringe sharing was lower than previously reported [9], which may be due in part to country-wide responses to HIV by increasing the levels of syringe distribution [3–6, 9].

Though most PWID (>90 %) were recruited from harm reduction sites, pharmacies, rather than these sites, were the main source of obtaining syringes. Pharmacies appear to be a critical and highly accessible source of syringes, although 18.6 % of participants reported that they had experienced a refusal to their request to purchase syringes. Almost all PWID did not dispose of their used syringes safely [45]. Though eight of 16 PDHRS were available for PWID, but only at low level (<20 %), the acceptance rates were high, and PWID were highly supportive of additional PDHRS.

Not surprising, free syringes from pharmacies are not currently offered. The success of NPSS is as part of the pharmacy business. Although NPSS from pharmacies were common, operation hours created a significant barrier against purchasing syringes. Perceived discrimination may also prevent PWID from purchasing syringes from pharmacies [30]. In other studies in Yunnan, PWID reported pretending to buy OTC drugs when purchasing syringes to avoid discrimination [41, 42]. In this study, most PWID wanted to buy syringes from pharmacies where they buy OTC or prescription drugs, but less than half chose pharmacies at the same neighborhood/region where they live. These findings suggest that fears of discrimination and stigma either from pharmacists/pharmacy staff or from others in their neighborhood are potential barriers against obtaining syringes from pharmacies. Prior research has also found that hostile service environments may increase the odds of improper syringe disposal [46].

Free printed materials about HIV, Hepatitis C, and STI risk reduction and free blood pressure checks that are commonly used to promote business ranked the first and second most frequently used PDHRS. The other six PDHRS targeting high risk populations were provided by less than 3 % of pharmacies. Future pharmacy based harm reduction interventions could begin with services that are mutually beneficial to pharmacies and PWID. Naloxone [47] would be an example of such service.

There were eight categories of services not currently offered in pharmacies in Xichang. Lack of “free condoms”, “free syringes”, and “exchanging used syringes for new ones” is probably linked with business concerns of reduced revenue for these services [18, 29–31, 35–37]. Legal considerations [23] may contribute to the lack of HIV testing [48–50], vaccinations for Hepatitis A and B [51], syringe disposal [45], and directly dispensing methadone [47, 52]. The dearth of “a written drug treatment referral” may be due to lack of “time” and professional training for pharmacists [18, 29–31, 35, 37]. Financial concerns could be resolved by reimbursements, similar to pharmacy vouchers, for syringes [19]. At the time of investigation, HIV testing was only available in medical facilities [48–50] not pharmacies. After approval from China FDA, HIV test kits may become available in pharmacies [48]. Currently, methadone clinics are only government administered [7]. As an alternative, pharmacies in areas with high numbers of PWID could dispense methadone [7, 52, 53], which may make MMT more available and cost-effective than only dispensing at national clinics [7] where discrimination and stigma have been documented [19, 20, 22, 23, 54]. Similarly, used syringes could be disposed at pharmacies [46]. In order to offer greater harm reduction services, pharmacy staff need training to improve their knowledge and skills (including drug treatment referrals) and enhanced sensitivity toward PWID. Due to easy access at community health centers [49–51], it is not necessary to provide vaccinations in pharmacies.

The limitations of the current study should be noted. All data was based on self-reports. Snowball sampling was used, and the participants were recruited primarily from harm reduction sites limiting the generalizability of the findings. Future studies should recruit PWID from the general communities. Respondent driven sampling could be applied [55]. Further studies should also examine barriers for pharmacies to provide harm reduction services.

## Conclusions

Although community pharmacies have been providing services to PWID in many countries and in parts of China, comprehensive PDHRS had not been reported in Liangshan where HIV is prevalent and the major route of HIV infection is injection drug use. The findings indicated that NPSS from pharmacies provided most PWID with new syringes, but disposal of used syringes was problematic. At the time of investigation, 8 of 16 services were already available. PWID were interested in using all the potential PDHRS, if available. Of the 16 PDHRS, 14 were feasible to be provided if pharmacies benefited financially from providing such services. Future harm risk reduction programs should provide the reimbursements for pharmacies and training for pharmacists.
